# Characterization and Management of Adverse Reactions From the CLEAR Study in Advanced Renal Cell Carcinoma Treated With Lenvatinib Plus Pembrolizumab

**DOI:** 10.1093/oncolo/oyac269

**Published:** 2023-03-02

**Authors:** Robert Motzer, Saby George, Jaime R Merchan, Thomas E Hutson, Xun Song, Rodolfo F Perini, Ran Xie, Urmi Bapat, Javier Puente

**Affiliations:** Department of Medicine, Memorial Sloan Kettering Cancer Center, New York, NY, USA; Department of Medicine, Roswell Park Comprehensive Cancer Center, Buffalo, NY, USA; Department of Medicine, University of Miami Sylvester Comprehensive Cancer Center, Miami, FL, USA; Department of Medical Oncology, Texas Oncology-Baylor Charles A. Sammons Cancer Center, Dallas, TX, USA; Clinical Research, Merck & Co., Inc., Rahway, NJ, USA; Clinical Research, Merck & Co., Inc., Rahway, NJ, USA; Biostatistics, Eisai Inc., Nutley, NJ, USA; Clinical Research, Eisai Inc., Nutley, NJ, USA; Department of Medical Oncology, Hospital Clinico Universitario San Carlos, Madrid, Spain

**Keywords:** adverse reactions, lenvatinib, pembrolizumab, renal cell carcinoma, management of adverse reactions

## Abstract

**Background:**

Lenvatinib plus pembrolizumab showed significantly improved progression-free and overall survival outcomes compared with sunitinib in patients with advanced renal cell carcinoma in the CLEAR study (NCT02811861). Here, we used CLEAR data to characterize common adverse reactions (ARs; adverse-event preferred terms grouped in accordance with regulatory authority review) associated with lenvatinib plus pembrolizumab and review management strategies for select ARs.

**Materials and Methods:**

Safety data from the 352 patients who received lenvatinib plus pembrolizumab in the CLEAR study were analyzed. Key ARs were chosen based on frequency of occurrence (≥30%). Time to first onset and management strategies for key ARs were detailed.

**Results:**

The most frequent ARs were fatigue (63.1%), diarrhea (61.9%), musculoskeletal pain (58.0%), hypothyroidism (56.8%), and hypertension (56.3%); grade ≥3 severity ARs that occurred in ≥5% of patients were hypertension (28.7%), diarrhea (9.9%), fatigue (9.4%), weight decreased (8.0%), and proteinuria (7.7%). Median times to first onset of all key ARs were within approximately 5 months (approximately 20 weeks) of starting treatment. Strategies for effectively managing ARs included baseline monitoring, drug–dose modifications, and/or concomitant medications.

**Conclusion:**

The safety profile of lenvatinib plus pembrolizumab was consistent with the known profile of each monotherapy; ARs were considered manageable with strategies including monitoring, dose modifications, and supportive medications. Proactive and prompt identification and management of ARs are important for patient safety and to support continued treatment.

**Clinicaltrials.gov ID:**

NCT02811861

## Teaser

This study used data from the CLEAR study to characterize common adverse reactions associated with lenvatinib plus pembrolizumab. This article reports management strategies for select adverse reactions.

**Implications for Practice**

Lenvatinib plus pembrolizumab has shown efficacy in the treatment of patients with previously untreated advanced renal cell carcinoma. Patients may experience adverse reactions associated with this combination, including fatigue, diarrhea, musculoskeletal pain, hypothyroidism, hypertension, stomatitis, decreased appetite, rash, nausea, dysphonia, proteinuria, and weight decreased. It is critical that the clinical team monitors and promptly identifies adverse reactions in patients to implement management strategies including dose modifications and supportive care. This article describes characteristics of common adverse reactions in patients receiving lenvatinib plus pembrolizumab and provides guidance for their identification and effective management.

## Introduction

Lenvatinib, a multitargeted tyrosine kinase inhibitor (TKI), plus pembrolizumab, an anti-programmed death-1 (anti-PD-1) antibody and immune checkpoint inhibitor (ICI), significantly improved efficacy outcomes versus sunitinib in patients with advanced renal cell carcinoma (aRCC) in the CLEAR study (Clinicaltrials.gov identifier: NCT02811861)^1^: median progression-free survival, as assessed by independent review committee, was significantly longer with lenvatinib plus pembrolizumab (23.9 months) versus sunitinib (9.2 months; hazard ratio [HR] 0.39, 95% CI, 0.32–0.49; *P* < .001); overall survival was also significantly longer with lenvatinib plus pembrolizumab versus sunitinib (HR 0.66, 95% CI, 0.49–0.88; *P* = .005). Objective response rate, as assessed by independent review committee, was greater with lenvatinib plus pembrolizumab (71.0%) versus sunitinib (36.1%; relative risk 1.97, 95% CI, 1.69–2.29).^1^ Based on the results of the CLEAR study, lenvatinib plus pembrolizumab was approved by the United States Food and Drug Administration (FDA) and the European Medicines Agency for the first-line treatment of adult patients with aRCC in 2021.^2,3^

The adverse event profile of lenvatinib plus pembrolizumab was considered manageable and generally consistent with the established profiles of each monotherapy.^1,2,4^ This post hoc analysis characterizes adverse reactions (ARs) that were observed in patients treated with the combination of lenvatinib plus pembrolizumab in the CLEAR study. Per the FDA, ARs are grouped terms of adverse events that are reasonably associated with the use of a drug.^5^ Factors used in determining the association of adverse events with the use of a drug include: frequency of reporting, comparison of adverse-event rate with drug versus placebo, extent of dose–response, extent to which an adverse event is consistent with the pharmacology of the drug, timing of the adverse event relative to time of drug exposure, existence of challenge and dechallenge experience, and whether the adverse event is known to be caused by related drugs in the same class. Notably, adverse events that are reported under different terms but represent the same phenomena are grouped together as a single AR, to avoid diluting or obscuring the true effect.^5^ The ARs associated with lenvatinib plus pembrolizumab from the CLEAR study are listed in the respective lenvatinib and pembrolizumab prescribing information,^2,4^ and specific information regarding immune-related ARs (irARs) related to pembrolizumab are discussed in the pembrolizumab prescribing information and prior publications.^4,6^ It is critical for a clinical team to promptly recognize and manage ARs for patient safety and to foster dose adjustment and supportive measures to extend time on therapy and resulting treatment benefits. The objective of the analyses presented here is to specifically characterize common ARs in previously untreated patients with aRCC who were treated with the combination of lenvatinib plus pembrolizumab^2-4^ in the CLEAR study. We also discuss optimal management strategies for patients with selected ARs treated with this combination.

## Materials and Methods

### Patients and Study Design

Patients with aRCC were randomly assigned to receive lenvatinib at a starting dose of 20 mg orally once daily and pembrolizumab at 200 mg intravenously every 3 weeks. The primary study report, including other eligibility criteria, has been published previously.^1^ This analysis focused on the characterization and management of ARs in patients with aRCC who had received at least 1 dose of study drug by the data cutoff date of August 28, 2020 (median overall survival follow-up: 26.6 months).

### Adverse Reactions

The FDA prescribing information for lenvatinib and pembrolizumab^2,4^ pool different adverse-event preferred terms that represent the same phenomena into grouped terms called ARs. ARs are indication and drug-combination specific and, per their regulatory definition, are considered to be reasonably associated with treatments, although a definitive causal relationship may not be established.^5^ In this analysis, key ARs were chosen based on frequency of occurrence (in ≥30% of patients)^2^ and preferred terms included in each key AR are shown in Table 1. ARs could have occurred while receiving lenvatinib and/or pembrolizumab or within the protocol-defined follow-up period of 30 days after the patient’s last dose; ARs were recorded until the end of the follow-up period or until resolution, whichever came first. Grading of ARs was performed according to Common Terminology Criteria for Adverse Events v4.03 (Supplementary Table S1). Additional methods for exposure-adjusted ARs are included in the Supplementary Appendix.

**Table 1.** Preferred terms included in each adverse reaction.^2^

Adverse reaction	Preferred terms
Fatigue	Fatigue, asthenia, malaise, and lethargy
Diarrhea	Diarrhea and gastroenteritis
Musculoskeletal pain	Arthralgia, arthritis, back pain, bone pain, breast pain, musculoskeletal chest pain, musculoskeletal discomfort, musculoskeletal pain, musculoskeletal stiffness, myalgia, neck pain, noncardiac chest pain, pain in extremity, and pain in jaw
Hypothyroidism	Hypothyroidism, increased blood thyroid-stimulating hormone, and secondary hypothyroidism
Hypertension	Essential hypertension, increased blood pressure, increased diastolic blood pressure, hypertension, hypertensive crisis, hypertensive retinopathy, and labile blood pressure
Stomatitis	Aphthous ulcer, gingival pain, glossitis, glossodynia, mouth ulceration, mucosal inflammation, oral discomfort, oral mucosal blistering, oral pain, oropharyngeal pain, pharyngeal inflammation, and stomatitis
Decreased appetite	Decreased appetite and early satiety
Weight decreased	Weight decreased
Rash	Genital rash, infusion site rash, penile rash, rash, rash erythematous, rash macular, rash maculo-papular, rash papular, rash pruritic, and rash pustular
Nausea	Nausea
Dysphonia	Dysphonia
Proteinuria	Hemoglobinuria, nephrotic syndrome, and proteinuria

## Results

### Patients

From the 1069 patients randomly assigned to a treatment in the CLEAR study, 355 were assigned to receive lenvatinib plus pembrolizumab of which 352 were treated with lenvatinib plus pembrolizumab.^1^ Baseline demographics and disease characteristics of the patients were balanced between groups and have been previously reported.^1^

### Key Adverse Reactions

**Figure 1.**
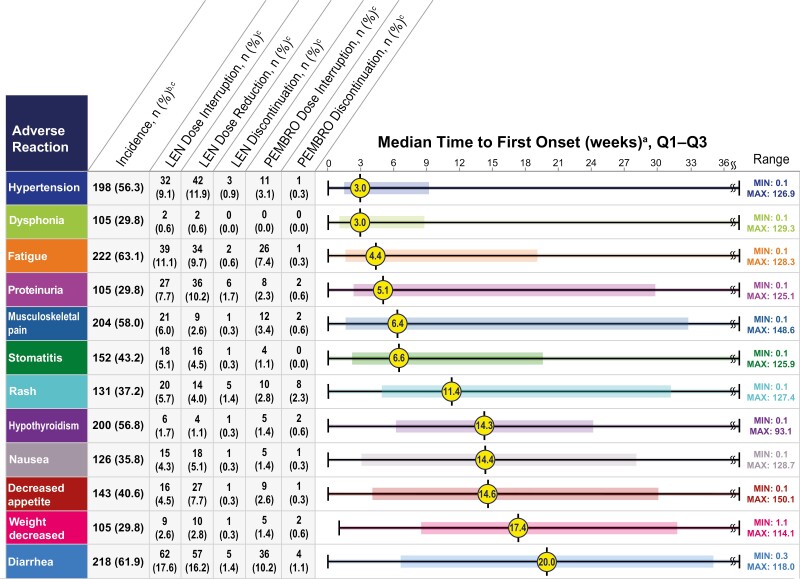
Median time to first onset of key adverse reactions (any grade) and dose management. ^a^Median time to first onset in patients who experienced the adverse reaction. Colored boxes represent Q1–Q3. Lines represent the range. ^b^Any grade. ^c^Percentages are based on the safety population of the lenvatinib + pembrolizumab group (*n* = 352). The safety population included all patients who received at least 1 dose of any study drug. LEN, lenvatinib; max, maximum; min, minimum; PEMBRO, pembrolizumab.

**Figure 2.**
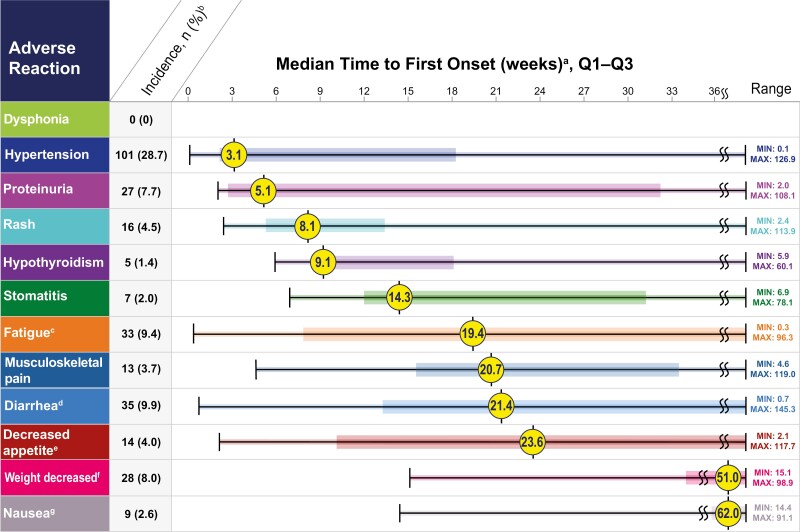
Median time to first onset of key adverse reactions (grade ≥3). ^a^Median time to first onset in patients who experienced the grade ≥3 adverse reaction. Colored boxes represent Q1–Q3. Lines represent the range. ^b^Any grade. Percentages are based on the safety population of the lenvatinib + pembrolizumab group (*n* = 352). The safety population included all patients who received at least 1 dose of any study drug. ^c^Q1 = 7.86; Q3 = 42.29. ^d^Q1 = 13.29; Q3 = 56.71. ^e^Q1 = 10.14; Q3 = 69.14. ^f^Q1 = 34.00; Q3 = 64.71. ^g^Q1 = 42.57; Q3 = 74.00. Max, maximum; Min, minimum; Q1, first quartile; Q3, third quartile.

Key ARs (incidence ≥30%, any grade) in the lenvatinib plus pembrolizumab group, regardless of causality, included fatigue (63.1%), diarrhea (61.9%), musculoskeletal pain (58.0%), hypothyroidism (56.8%), hypertension (56.3%), stomatitis (43.2%), decreased appetite (40.6%), rash (37.2%), nausea (35.8%), dysphonia (29.8%), proteinuria (29.8%), and weight decreased (29.8%) (Table 2). Further information regarding these key ARs is included in relevant sections below. The prescribing information for lenvatinib and pembrolizumab should be consulted for other important but less common ARs that may occur during treatment with lenvatinib plus pembrolizumab.^2,4^ Clinically relevant ARs (<20%) that occurred in patients receiving lenvatinib plus pembrolizumab were myocardial infarction (3%) and angina pectoris (1%).^2^ When adjusted for exposure (defined in Supplementary Appendix), the most frequent of the key ARs were diarrhea, musculoskeletal pain, fatigue, and hypertension (Table 3). Median times to first onset of key ARs and associated dose modifications are shown in Fig. 1; and median times to first onset of key ARs of grade ≥3 severity during lenvatinib plus pembrolizumab treatment are shown in Fig. 2.

**Table 2.** Adverse reactions with incidence ≥30% of patients in the lenvatinib-plus-pembrolizumab group.

Adverse reaction, %	Lenvatinib + pembrolizumab group (*n* = 352)^a^	
	Any grade	Grade ≥3
Fatigue	63.1	9.4
Diarrhea	61.9	9.9
Musculoskeletal pain	58.0	3.7
Hypothyroidism	56.8	1.4
Hypertension	56.3	28.7
Stomatitis	43.2	2.0
Decreased appetite	40.6	4.0
Rash	37.2	4.5
Nausea	35.8	2.6
Dysphonia	29.8	0
Proteinuria	29.8	7.7
Weight decreased	29.8	8.0

^a^All safety analyses included patients who received at least 1 dose of any study drug.

**Table 3.** Exposure-adjusted incidence of key adverse reactions.

Parameter	Lenvatinib + pembrolizumab group
Patients exposed, *n*	352
Total exposure^a^, person-years	524.9
Adverse reaction, total episodes^b^ (total episodes/total exposure)	
Diarrhea	567 (1.08)
Musculoskeletal pain	480 (0.91)
Fatigue	370 (0.70)
Hypertension	340 (0.65)
Hypothyroidism	249 (0.47)
Stomatitis	241 (0.46)
Decreased appetite	220 (0.42)
Nausea	218 (0.42)
Rash	199 (0.38)
Proteinuria	197 (0.38)
Dysphonia	134 (0.26)
Weight decreased	125 (0.24)

^a^Drug exposure was defined as 1 day added to the interval (converted to years) between the first dose date and the earlier of the last dose date + 30 days or the database cutoff date + 1 day. Total exposure is the sum of drug exposure for all patients in treatment group (including dose interruption).

^b^Total number of episodes: a single episode is defined from onset through resolution or, if ongoing, to the end of reporting period.

#### General Patient-Management Strategies for ARs

**Figure 3.**
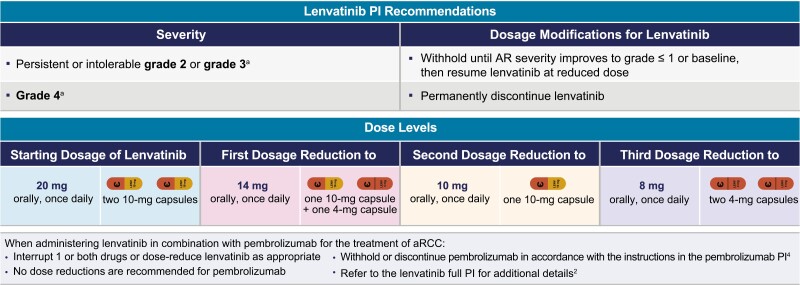
Management guidelines for adverse reactions according to the US FDA lenvatinib prescribing information. aPlease note there are exceptions to the grade 3- and grade 4-severity advice; some grade 3 ARs require treatment discontinuation, whereas some grade 4 ARs do not. bPer the CLEAR study protocol, lenvatinib should be withheld until AR severity improves to tolerable grade 2 or grade ≤1. In the CLEAR study, among patients who received lenvatinib plus pembrolizumab (n = 352), 106 (30.1%) patients had 1 dose reduction, 82 (23.3%) patients had 2 dose reductions, 42 (11.9%) had 3 dose reductions, and 19 (5.4%) patients had 4 dose reductions. Note the bullet point instructions for administering lenvatinib in combination with pembrolizumab for the treatment of aRCC.2,4. Abbreviations: AR, adverse reaction; aRCC, advanced renal cell carcinoma; PI, prescribing information.

Early and proactive approaches are important to optimally manage ARs in patients treated with lenvatinib plus pembrolizumab. Clinicians should diligently educate themselves and the treating team regarding common ARs associated with the combination treatment and also ensure proper education of patients and caregivers prior to starting treatment. Dose reductions and interruptions are important strategies for AR management, but when possible, it is suggested to utilize medical management before initiating dose modifications. For most of the key ARs related to lenvatinib, as indicated by the lenvatinib label, the management advice is generally to withhold lenvatinib for ARs of persistent or intolerable grade 2, or any grade 3, severity. Upon resolution to grade ≤1 severity or baseline AR level, lenvatinib dosage can be reduced progressively to 14 mg, 10 mg, and 8 mg, each step once daily; permanent discontinuation of lenvatinib is recommended for most grade 4 severity ARs (Fig. 3).^2^ Management advice from the CLEAR study protocol,^1^ while similar to the prescribing information, allowed patients to resume lenvatinib at a reduced dose level on resolution of most ARs (unless noted otherwise) to tolerable grade 2 or grade ≤1 severity (Fig. 3).

Dose reduction for pembrolizumab is not recommended.^4^ For most irARs associated with pembrolizumab of grade 3 or intolerable grade 2 severity, treatment should be withheld and then resumed after the AR has improved to grade 0 or 1 severity and any corticosteroid dose has been tapered. Pembrolizumab should be permanently discontinued for life-threatening grade 4 severity irARs, recurrent severe (grade 3) irARs that require systemic immunosuppressive treatment, or if the irAR does not resolve within 12 weeks of last dose or corticosteroid doses cannot be reduced to ≤10 mg prednisone or equivalent per day within 12 weeks. Corticosteroid tapering should be initiated when the irAR improves to grade 0 or 1 severity and should continue over at least 4 weeks. For severe and life-threatening irARs, intravenous corticosteroids should be initiated first, followed by oral steroids. Other immunosuppressive treatment should be initiated if irARs cannot be controlled by corticosteroids.^1,4^ Further information regarding management of ARs related to pembrolizumab is included in the pembrolizumab label.^4^

Specific recommendations for hypertension, diarrhea, hypothyroidism, and proteinuria from the CLEAR study protocol are described in relevant sections below. Dose modifications were used as necessary^1^ and an overview is provided in the Supplementary Appendix.

#### Fatigue

The median time to the first onset of any-grade fatigue was 4.4 weeks and for grade ≥3 severity fatigue, 19.4 weeks (Figs. 1 and 2). No grade 4 or grade 5 ARs of fatigue were reported and patient incidences of fatigue by worst-grade severity were: any grade, 63.1%; grade 1, 29.5%; grade 2, 24.1%; and grade 3, 9.4% (Table 2). Due to fatigue, 11.1% of patients experienced lenvatinib interruptions, 7.4% experienced pembrolizumab interruptions, and 9.7% experienced lenvatinib dose reductions, while 0.6% of patients discontinued lenvatinib and 0.3% discontinued pembrolizumab (Fig. 1).

#### Diarrhea

The median time to first onset of any-grade diarrhea was 20.0 weeks and for grade ≥3 severity diarrhea, 21.4 weeks (Figs. 1 and 2). No grade 4 or grade 5 ARs of diarrhea were reported and patient incidences of diarrhea by worst-grade severity were: any grade, 61.9%; grade 1, 23.9%; grade 2, 28.1%; and grade 3, 9.9% (Table 2). Per the CLEAR study protocol, an antidiarrheal agent was recommended to patients at the start of study treatment and patients were instructed and educated to initiate antidiarrheal treatment at the first onset of any soft bowel movements.^1^ Patients should be advised to drink liberal quantities of clear fluids.^1^ If sufficient oral fluid intake is not feasible, fluid and electrolytes should be substituted via intravenous infusion, per the CLEAR study protocol.^1^ Prompt management of diarrhea is recommended—if diarrhea persists despite medical management, then lenvatinib treatment should be withheld and resumed at a lower dose upon recovery or permanently discontinued (based on severity).^2^ Diarrhea could be caused by either lenvatinib or pembrolizumab treatment, so if dose interruption of lenvatinib does not lead to clinical improvement, patients should be monitored for symptoms of enterocolitis (ie, diarrhea, abdominal pain, blood or mucus in stool, and with or without fever) and bowel perforation (ie, peritoneal signs and ileus), per the study protocol.^1^ Patients who have grade ≥2 severity diarrhea with suspected colitis should consider a consultation with a gastrointestinal specialist for an endoscopy to assess for immune-related colitis.^1^ Pembrolizumab treatment should be withheld for grade 2 or 3 severity diarrhea/colitis and should be permanently discontinued if the severity is of grade 4.^1,4^ Corticosteroids (initial dose of 1–2 mg/kg prednisone or equivalent) should be administered followed by tapering doses. In case of no clinical improvement, additional immunosuppressants may be used to treat colitis. In CLEAR, due to diarrhea, 17.6% of patients experienced lenvatinib interruptions, 10.2% experienced pembrolizumab interruptions, and 16.2% experienced lenvatinib dose reductions, while 1.4% of patients discontinued lenvatinib and 1.1% discontinued pembrolizumab (Fig. 1).

#### Musculoskeletal Pain

The median time to first onset of any-grade musculoskeletal pain was 6.4 weeks and for grade ≥3 severity musculoskeletal pain, 20.7 weeks (Figs. 1 and 2). No grade 4 or grade 5 ARs were reported and patient incidences of musculoskeletal pain by worst-grade severity were: any grade, 58.0%; grade 1, 33.0%; grade 2, 21.3%; and grade 3, 3.7%. Due to musculoskeletal pain, 6.0% of patients experienced lenvatinib interruptions, 3.4% experienced pembrolizumab interruptions, and 2.6% experienced lenvatinib dose reductions, while 0.3% of patients discontinued lenvatinib and 0.6% discontinued pembrolizumab.

#### Hypothyroidism

The median time to first onset of any-grade hypothyroidism was 14.3 weeks and for grade ≥3 severity hypothyroidism, 9.1 weeks (Figs. 1 and 2). No grade 4 or grade 5 severity ARs of hypothyroidism were reported and patient incidences of hypothyroidism by worst-grade severity were: any grade, 56.8%; grade 1, 14.5%; grade 2, 40.9%; and grade 3, 1.4% (Table 2). Per the lenvatinib prescribing information, thyroid function should be monitored prior to initiating treatment and at least monthly during treatment^2^; per the CLEAR study protocol, thyroid function was monitored every 2 treatment cycles.^1^ Hypothyroidism should be treated according to standard medical practice per the lenvatinib label.^2^ Hypothyroidism has been reported with both lenvatinib and pembrolizumab as monotherapies in prior studies,^2,4^ and endocrine abnormalities have been reported in association with immunotherapies,^7^ thus warranting vigilance for endocrine-related ARs upon treatment with lenvatinib plus pembrolizumab. The pembrolizumab label recommends initiating hormone replacement therapy for hypothyroidism and withholding treatment for grades 3 or 4 severity endocrinopathies until patients are clinically stable or permanently discontinuing, depending on severity.^4^ The CLEAR study protocol recommended management of grades 2–4 severity hypothyroidism by continuing pembrolizumab treatment, initiating thyroid replacement hormones (eg, levothyroxine or liothyronine) per standard of care, and monitoring for symptoms of thyroid disorders.^1^ Minimal dose modifications to treat hypothyroidism were used, with 1.7% of patients experiencing lenvatinib interruptions, 1.4% experiencing pembrolizumab interruptions, and 1.1% experiencing lenvatinib dose reductions, while 0.3% of patients discontinued lenvatinib and 0.6% discontinued pembrolizumab.

#### Hypertension

The median time to first onset of any-grade hypertension was 3.0 weeks and for grade ≥3 severity hypertension, 3.1 weeks (Figs. 1 and 2). Patient incidences of hypertension by worst-grade severity were: any grade, 56.3%; grade 1, 6.5%; grade 2, 21.0%; grade 3, 28.1%; grades 4 and 5, 0.3% each (Table 2). The CLEAR study protocol required eligible patients to have a blood pressure (BP) of ≤150/90 mm Hg at the time of study entry and, if known to be hypertensive, patients should have been on a stable dose of antihypertensive therapy for at least 1 week before cycle 1/day 1.^1^ Hypertension was graded based only on BP measurements and not on the number of antihypertensive medications; per protocol, BP was monitored before beginning treatment (screening and baseline) and on days 1 and 15 of cycles 1 and 2.^1^ Thereafter, BP was monitored on day 1 of each cycle; patients with systolic BP ≥160 mm Hg or diastolic BP ≥100 mm Hg had their BP monitored on day 15 (or more frequently as clinically indicated) until systolic BP was ≤150 mm Hg and diastolic BP was ≤95 mm Hg for 2 consecutive treatment cycles. Dose modifications were used as necessary to treat hypertension, with 9.1% of patients experiencing lenvatinib interruptions, 3.1% experiencing pembrolizumab interruptions, and 11.9% experiencing lenvatinib dose reductions, while 0.9% of patients discontinued lenvatinib and 0.3% discontinued pembrolizumab (Fig. 1). Per the lenvatinib label, BP should be controlled prior to treatment and monitored during treatment; lenvatinib should be withheld for patients experiencing grade 3 hypertension despite optimal antihypertensive therapy, but treatment can be resumed at a reduced dose when hypertension is controlled at grade 2 or lower severity.^2^ Lenvatinib should be withheld in any instance where a patient is at imminent risk of developing a hypertensive crisis or has significant risk factors for severe complications of uncontrolled hypertension^1^ and should be permanently discontinued if hypertension reaches grade 4 severity.^2^ Further details regarding management of hypertension are included in the Supplementary Appendix.

#### Stomatitis

The median time to first onset of any-grade stomatitis was 6.6 weeks and for grade ≥3 severity, 14.3 weeks (Figs. 1 and 2). No grade 4 or grade 5 ARs of stomatitis were reported and patient incidences of stomatitis by worst-grade severity were: any grade, 43.2%; grade 1, 27.0%; grade 2, 14.2%; and grade 3, 2.0% (Table 2). Due to stomatitis, 5.1% of patients experienced lenvatinib interruptions, 1.1% experienced pembrolizumab interruptions, and 4.5% experienced lenvatinib dose reductions, while 0.3% of patients discontinued lenvatinib and no patients discontinued pembrolizumab (Fig. 1).

#### Decreased Appetite and Weight Decreased

The median times to first onset of any-grade decreased appetite and weight decreased were 14.6 weeks (grade ≥3, 23.6 weeks) and 17.4 weeks (grade ≥3, 51.0 weeks), respectively (Figs. 1 and 2). No grade 4 or grade 5 ARs were reported and patient incidences of decreased appetite/weight decreased by worst-grade severity were: any grade, 40.6%/29.8%; grade 1, 20.5%/7.7%; grade 2, 16.2%/14.2%; and grade 3, 4.0%/8.0% (Fig. 1). To address decreased appetite/weight decreased, 4.5%/2.6% of patients experienced lenvatinib interruptions, 2.6%/1.4% experienced pembrolizumab interruptions, and 7.7%/2.8% experienced lenvatinib dose reductions, while 0.3%/0.3% of patients discontinued lenvatinib and 0.3%/0.6% discontinued pembrolizumab (Fig. 1).

#### Rash

The median time to first onset of any-grade rash was 11.4 weeks and for grade ≥3 severity, 8.1 weeks (Figs. 1 and 2). No grade 4 or grade 5 severity ARs of rash were reported and patient incidences of rash by worst-grade severity were: any grade, 37.2%; grade 1, 22.4%; grade 2, 10.2%; and grade 3, 4.5% (Table 2). Due to rash, 5.7% of patients experienced lenvatinib interruptions, 2.8% experienced pembrolizumab interruptions, and 4.0% experienced lenvatinib dose reductions, while 1.4% of patients discontinued lenvatinib and 2.3% discontinued pembrolizumab (Fig. 1).

#### Nausea

The median time to first onset of any-grade nausea was 14.4 weeks and for grade ≥3 severity, 62.0 weeks (Figs. 1 and 2). No grade 4 or grade 5 severity ARs of nausea were reported and patient incidences of nausea by worst-grade severity were: any grade, 35.8%; grade 1, 18.2%; grade 2, 15.1%; and grade 3, 2.6% (Table 2). Medical management of nausea was required before lenvatinib dose reductions.^1^ Due to nausea, 4.3% of patients experienced lenvatinib interruptions, 1.4% experienced pembrolizumab interruptions, and 5.1% experienced lenvatinib dose reductions, while 0.3% of patients discontinued lenvatinib and 0.3% discontinued pembrolizumab (Fig. 1).

#### Dysphonia

The median time to first onset of any-grade dysphonia was 3.0 weeks (Fig. 1). No grade 3 through 5 ARs of dysphonia were reported and patient incidences of dysphonia by worst-grade severity were: any grade, 29.8%; grade 1, 26.1%; and grade 2, 3.7% (Table 2). Due to dysphonia, 0.6% of patients experienced lenvatinib interruptions, 0.6% experienced lenvatinib dose reductions, and no patients experienced pembrolizumab interruptions, discontinued lenvatinib, or discontinued pembrolizumab (Fig. 1).

#### Proteinuria

The median time to first onset of any-grade and grade ≥3 severity proteinuria was 5.1 weeks each (Figs. 1 and 2). No grade 4 or grade 5 severity ARs of proteinuria were reported and patient incidences of proteinuria by worst-grade severity were: any grade, 29.8%; grade 1, 6.0%; grade 2, 16.2%; and grade 3, 7.7% (Table 2). Monitoring proteinuria prior to initiation of treatment and regular monitoring during treatment thereafter are recommended.^2^ In CLEAR, urine dipstick testing for patients with proteinuria ≥2+ was performed on day 15 (or more frequently as clinically indicated) until the results were 1+ or negative for 2 consecutive treatment cycles. Lenvatinib should be withheld for ≥2 g proteinuria per 24 h, and resumed at a lower dose when proteinuria is ≤2 g per 24 h.^2^ Treatment should be discontinued for nephrotic syndrome.^2^ Further strategies for proteinuria monitoring and management are included in the Supplementary Appendix. Due to proteinuria, 7.7% of patients experienced lenvatinib interruptions, 2.3% experienced pembrolizumab interruptions, and 10.2% experienced lenvatinib dose reductions, while 1.7% of patients discontinued lenvatinib and 0.6% discontinued pembrolizumab (Fig. 1).

### Concomitant Medications

In the CLEAR study, most patients received at least 1 concomitant medication, although the exact reasons for medications were not summarized (Supplementary Table 2). Details regarding high-dose corticosteroids are included in the Supplementary Appendix.

## Discussion

Data from recent clinical trials emphasize the importance of combination therapies (TKI + ICI, ICI + ICI), which have shown notably improved efficacy outcomes in patients with renal cell carcinoma; these therapies are also associated with distinct safety profiles.^1,2,4,8-15^ ICI + ICI combination therapies are associated with specific immune-mediated adverse events that include gastrointestinal, endocrine, dermatologic, and pulmonary adverse events.^8,9^ TKIs are known to be associated with certain adverse events (eg, fatigue, asthenia, diarrhea, nausea, anorexia, rash, hand-foot syndrome, hypertension) when used in the treatment of patients with metastatic renal cell carcinoma.^16^ Treatment with TKI + ICI combination therapies also may be associated with a higher risk of diarrhea and decreased appetite.^17^

To ensure patient safety, healthcare providers should educate themselves on the patient management modalities for ARs associated with each treatment program. Also, for lenvatinib therapy, several studies have indicated that the optimal dosing strategy across indications is to start treatment at the recommended doses, and then interrupt or dose reduce, as necessary, to optimally manage ARs while supporting continuation of treatment.^18-22^ For this strategy to be successful, active engagement of the multidisciplinary clinical team is crucial to ensure proactive and prompt identification and management of ARs. Specifically, baseline monitoring of blood pressure, urine protein levels, and thyroid and liver function prior to lenvatinib treatment are recommended^2^; moreover, regular monitoring of blood pressure, proteinuria, electrolytes, blood calcium levels, and thyroid and liver function are also recommended during treatment with lenvatinib.^2^ For most of the key ARs, the management advice^2^ is to withhold lenvatinib treatment for persistent or intolerable grade 2 and all grade 3 severity. Upon resolution to grade ≤1 or baseline severity (or to tolerable grade 2 per the CLEAR study protocol), lenvatinib treatment can be resumed at a lower dose. For most ARs, if the severity reaches grade 4, it is recommended to permanently discontinue lenvatinib; in general, pembrolizumab should be discontinued for grade 4 immune-mediated ARs.^2,4^ Optimal medical management should be used when available and applicable prior to lenvatinib dose reduction (eg, for nausea, hypertension, diarrhea, hypothyroidism); lenvatinib and/or pembrolizumab dose interruptions or lenvatinib dose reductions should be initiated according to the respective product prescribing information. Dosing interventions, including dosing interruptions for lenvatinib and pembrolizumab and dose reductions for lenvatinib, are important management strategies for ARs, and lenvatinib dose modifications were judiciously used in the CLEAR trial to manage ARs as appropriate.^1^

Certain ARs (eg, hypothyroidism, diarrhea) may be attributable to either lenvatinib or pembrolizumab at first onset. Since management strategies differ, it is important to determine the causative agent for such toxicities versus an alternative etiology. The timing of first onset of ARs and AR resolution may be informative when contextualized against the facts that lenvatinib has a shorter half-life (28 h) and is administered daily.^2^ Dose interruption of lenvatinib may be considered a first-line approach to determine if clinical resolution can be reached. In case of no clinical improvement, an immune-mediated AR should be considered. Per the pembrolizumab label, known irARs include pneumonitis, colitis, hepatitis, endocrinopathies, nephritis with renal dysfunction, dermatologic irARs, and solid-organ transplant rejection.^4^ If ARs discussed here are considered to be attributable to pembrolizumab, or in the case of known irARs, physicians should refer to the pembrolizumab prescribing information for appropriate management strategies. For colitis, additional agents (like infliximab or vedolizumab) may be used in addition or in place of steroid treatments, pending recommendations of each clinic. Severe ARs may sometimes require interruption of both study drugs and initiation of concomitant medications.

This analysis of data from CLEAR indicated that ARs in patients treated with lenvatinib plus pembrolizumab therapy were consistent with the known profiles of each monotherapy. The median time to first onset of all key ARs in this analysis occurred within 5 months (approximately 20 weeks) of treatment initiation; however, clinicians should be vigilant in monitoring the development of ARs throughout treatment.

## Conclusions

Close monitoring of patients treated with lenvatinib plus pembrolizumab is critical because ARs can occur at any time and can be managed with additional medical therapy if they are diagnosed promptly. The clinical team plays a critical role in the identification and management of ARs in patients with aRCC treated with lenvatinib plus pembrolizumab. Prompt management of ARs may potentially reduce treatment interruption(s) and/or lenvatinib dose reduction and allow patients to continue receiving therapy.

## Supplementary Material

oyac269_suppl_Supplementary_Material

## Data Availability

The data will not be available for sharing at this time because the data are commercially confidential. However, Eisai Inc. will consider written requests to share the data on a case-by-case basis. R. Motzer (lead/corresponding author) confirms that he had full access to all the data in the study and takes responsibility for the integrity of the data and the accuracy of the data analysis.
