# Supplementary material for: Characterization and Management of Adverse Reactions From the CLEAR Study in Advanced Renal Cell Carcinoma Treated With Lenvatinib Plus Pembrolizumab
Source: Oncologist. 2023 Mar 2;28(6):501–9. doi: 10.1093/oncolo/oyac269 (PMC10243770; doi:10.1093/oncolo/oyac269)
Supplement: oyac269_suppl_Supplementary_Material [file oyac269_suppl_supplementary_material.docx]

**SUPPLEMENTARY APPENDIX**

**Methods:**

*Exposure-adjusted adverse reactions (ARs)*

Exposure-adjusted incidences of adverse reactions were calculated by dividing the total number of episodes (n) by total exposure (n/total exposure). Total exposure referred to the total duration of treatment (in years) among all patients. Based on exposure-adjusted incidence rates, the most frequent key ARs included any for which n/total exposure >0.6.

**Results:**

*Overview of dose modifications in the CLEAR study*

Due to an AR, dose interruptions of lenvatinib, pembrolizumab, or both, occurred in 78% of patients receiving the combination therapy (lenvatinib, 73%; both drugs, 39%) [1]. Lenvatinib dose was reduced in 69% of patients [1]. Among patients who received lenvatinib plus pembrolizumab (n=352), 106 (30.1%) patients had 1 dose reduction, 82 (23.3%) patients had 2 dose reductions, 42 (11.9%) had 3 dose reductions, and 19 (5.4%) patients had 4 dose reductions. Due to an AR, permanent discontinuation of lenvatinib, pembrolizumab, or both, occurred in 37% of patients (lenvatinib, 26%; pembrolizumab, 29%; both, 13%) [1]. Median time to first dose interruption of lenvatinib was 4.14 months (range: 0.07–30.59); median time to first dose reduction of lenvatinib was 1.87 months (range: 0.10–37.98).

*Concomitant high-dose corticosteroids*

Of the 352 patients who received lenvatinib plus pembrolizumab, 14.8% concomitantly received high-dose corticosteroids (≥40 mg prednisone daily equivalent) to manage immune-mediated adverse events [2]. High-dose corticosteroids were taken by 18 (5.1%) and 6 (1.7%) patients for ≥14 days and ≥30 days consecutively, respectively. Of the 355 patients randomly assigned

to receive lenvatinib plus pembrolizumab, 40.3% received at least 1 concomitant antidiarrheal medication and 82.3% received at least 1 dose of concomitant antihypertensive medication (**Supplementary Table 2**).

**Supplementary Table 1.** Selected adverse-event grades according to CTCAE v4.03

|  | **Grade 1** | **Grade 2** | **Grade 3** | **Grade 4** |
| --- | --- | --- | --- | --- |
| **Hypertension** | - Systolic BP 120-139 mm Hg or diastolic BP 80-89 mm Hg | - Systolic BP 140-159 mm Hg or diastolic BP 90-99 mm Hg - Medical intervention indicated - Recurrent or persistent (≥24 hrs) - Symptomatic increase by >20 mm Hg (diastolic) or to >140/90 mm Hg - Monotherapy indicated | - Systolic BP ≥160 mm Hg or diastolic BP ≥100 mm Hg - Medical intervention indicated - More than one drug or more intensive therapy than previously used indicated.^a^ | - Life-threatening consequences (eg, malignant hypertension, transient or permanent neurologic deficit, hypertensive crisis) - Urgent intervention indicated |
| **Fatigue** | - Fatigue relieved by rest | - Fatigue not relieved by rest - Limiting instrumental ADL | - Fatigue not relieved by rest - Limiting self-care ADL |  |
| **Nausea** | - Loss of appetite without alteration in eating habits | - Oral Intake decreased without significant weight loss, dehydration, or nutrition | - Inadequate oral caloric or fluid intake - Tube feeding or hospitalization indicated |  |
| **Vomiting** | - 1-2 episodes (separated by 5 minutes) in 24 h | - 3-5 episodes (separated by 5 minutes) in 24 h | - ≥6 episodes (separated by 5 minutes) in 24 h - Tube feeding, TPN, or hospitalization indicated | - Life-threatening consequences - Urgent intervention indicated |
| **Diarrhea** | - Increase of <4 stools per day over baseline - Mild increase in ostomy output compared with baseline | - Increase of 4–6 stools per day over baseline - Moderate increase in ostomy output compared with baseline | - Increase of ≥7 stools per day over baseline - Incontinence - Hospitalization indicated - Severe increase in ostomy output compared with baseline - Limiting self care ADL | - Life-threatening consequences - Urgent intervention indicated |
| **Anorexia^b^ (Decreased appetite)** | - Loss of appetite without alteration in eating habits | - Oral intake altered without significant weight loss or malnutrition - Oral nutritional supplements indicated | - Associated with significant weight loss or malnutrition (eg, inadequate oral caloric and/or fluid intake) - Tube feeding or TPN indicated | - Life-threatening consequences - Urgent intervention indicated |
| **Weight loss** | - 5 to <10% loss from baseline - Intervention not indicated | - 10 to <20% loss from baseline - Nutritional support indicated | - ≥20% loss from baseline - Tube feeding or TPN indicated |  |
| **Hypothyroidism** | - Asymptomatic - Clinical or diagnostic observations only - Intervention not indicated | - Symptomatic - Thyroid replacement indicated - Limiting instrumental ADL | - Severe symptoms - Limiting self care ADL - Hospitalization indicated | - Life-threatening consequences - Urgent intervention indicated |
| **PPES** | - Minimal skin changes or dermatitis (eg, erythema, edema, or hyperkeratosis) without pain | - Skin changes (eg, peeling, blisters, bleeding, fissures, edema, or hyperkeratosis) with pain - Limiting instrumental ADL | - Severe skin changes (eg, peeling, blisters, bleeding, fissures, edema, or hyperkeratosis) with pain - Limiting self care ADL |  |
| **Musculoskeletal pain** | - Mild pain | - Moderate pain - Limiting instrumental ADL | - Severe pain - Limiting self care ADL |  |
| **Oral mucositis^b^**  **(Stomatitis)** | - Asymptomatic or mild symptoms - Intervention not indicated | - Moderate pain; not interfering with oral intake - Modified diet indicated | - Severe pain; interfering with oral intake | - Life-threatening consequences - Urgent intervention indicated |
| **Proteinuria** | - 1+ proteinuria; urinary protein <1.0 g/24 h | - 2+ proteinuria; urinary protein 1.0-3.4 g/24 h | - Urinary protein ≥3.5 g/24 h |  |

Reference: CTCAE version 4.03, published June 14, 2010. <https://evs.nci.nih.gov/ftp1/CTCAE/CTCAE_4.03/CTCAE_4.03_2010-06-14_QuickReference_8.5x11.pdf>

^a^These criteria were not used in CLEAR. Hypertension was assessed by blood pressure only.

^b^Specific CTCAE grading is not available for the preferred term. The preferred term used in this analysis is included in parentheses.

ADL, activities of daily living; BP, blood pressure; CTCAE, Common Terminology Criteria for Adverse Events; PPES, palmar-plantar erythrodysesthesia syndrome; TPN, total parenteral nutrition; ULN, upper limit of normal.

**Supplementary Table 2:** Concomitant medications received by patients in the lenvatinib-plus-pembrolizumab group of the CLEAR study^a^

| Anatomical Class (ATC Level 1) Pharmacological Subclass (ATC Level 3), % | Lenvatinib + Pembrolizumab Group (n = 355) |
| --- | --- |
| **Patients with at least 1 concomitant medication** | 98.9 |
| **Patients with at least 1 concomitant medication (excluding antihypertensive, antidiarrheal medications, and corticosteroids for systemic use)** | 96.6 |
| **Antiinflammatory and antirheumatic products, nonsteroidal** | 31.8 |
| **Antithrombotic agents** | 40.8 |
| **Beta-lactam antibacterials, penicillins** | 27.3 |
| **Drugs for constipation** | 29.6 |
| **Drugs for peptic ulcer and gastroesophageal reflux disease (GERD)** | 58.6 |
| **Lipid-modifying agents, plain** | 3.4 |
| **Opioids** | 36.1 |
| **Other analgesics and antipyretics** | 54.4 |
| **Stomatological preparations** | 26.5 |
| **Thyroid preparations** | 54.1 |
| **Anatomical Class (ATC Level 1) WHO Drug Name Preferred Term), %** | **Lenvatinib + Pembrolizumab Group (n = 355)** |
| **Patients with at least 1 concomitant antihypertensive medication^b^** | 82.3 |
| **Amlodipine** | 41.1 |
| **Furosemide** | 15.8 |
| **Losartan** | 10.4 |
| **Bisoprolol** | 9.9 |
| **Patients with at least 1 concomitant antidiarrheal medication** | 40.3 |
| **Patients with at least 1 concomitant corticosteroid for systemic use** | 51.0 |

Data cutoff date: 28 August 2020.

^a^The exact reasons for concomitant medications were not summarized for most ARs.

^b^Medications listed below were the most common antihypertensive medications received by patients in the lenvatinib plus pembrolizumab group.

Percentages are based on the total number of patients in the Full Analysis Set within the relevant treatment group.

Concomitant medications include medications that either (1) started before the first dose of study drug and were continuing at the time of the first dose of study drug, or (2) started on or after the date of the first dose of study drug up to 30 days after the patient’s last dose.

Patients with 2 or more medications within an ATC level (or drug name) are counted only once within that ATC level (or drug name).

Medications were coded using WHO Drug Dictionary Version WHODDMAR20B3G.

ATC, Anatomical Therapeutic Chemical; WHO, World Health Organization.

**Management of adverse reactions [3]:**

*Hypertension:*

Hypertension is a recognized side effect of treatment with drugs inhibiting VEGF signaling [**4**]. Physicians should therefore ensure that patients receiving treatment with lenvatinib have blood pressure (BP) of ≤150/90 mm Hg before treatment starts and, if known to be hypertensive, have been on a stable dose of antihypertensive therapy for at least 1 week prior to treatment intitiation. Early detection and effective management of hypertension are important to minimize the need for lenvatinib dose interruptions and reductions.

Regular assessment of BP should be conducted. Hypertension is graded using CTCAE v4.03, based on BP measurements only (and not on the number of antihypertensive medications). Antihypertensive agents should be started as soon as elevated BP (systolic BP ≥140 mm Hg or diastolic BP ≥90 mm Hg) is confirmed on 2 assessments (at least 30 minutes later). The choice of antihypertensive treatment should be individualized to the patient’s clinical circumstances and follow standard medical practice. For previously normotensive patients, appropriate antihypertensive therapy should be started when systolic BP ≥140 mm Hg or diastolic BP ≥90 mm Hg is first observed on 2 assessments at least 30 minutes apart. For those patients already on antihypertensive medication, treatment modification may be necessary if hypertension persists.

Lenvatinib should be withheld in any instance where a patient is at imminent risk to develop a hypertensive crisis or has significant risk factors for severe complications of uncontrolled hypertension (eg, BP ≥160/100 mm Hg, significant risk factors for cardiac disease, intracerebral hemorrhage, or other significant co-morbidities). Once the patient has been on the same antihypertensive medications for at least 48 hours and the BP is controlled, lenvatinib should be resumed as described below. Patients with systolic BP ≥160 mm Hg or diastolic BP ≥100 mm Hg must have their BP monitored (as frequently as clinically indicated) until systolic BP has been ≤150 mm Hg and diastolic BP has been ≤95 mm Hg for 2 consecutive treatment cycles. If a repeat event of systolic BP ≥160 mm Hg or diastolic BP ≥100 mm Hg occurs, the patient must resume evaluation until systolic BP has been ≤150 mm Hg and diastolic BP has been ≤95 mm Hg for 2 consecutive treatment cycles. A diary should be provided to the patient to capture their BP evaluations outside of the office.

The following guidelines should be followed for the management of systolic BP ≥160 mmHg or diastolic BP ≥100 mmHg confirmed on repeat measurements after at least 30 minutes:

1. Continue lenvatinib and institute antihypertensive therapy for patients not already receiving this.

2. For those patients already on antihypertensive medication, the dose may be increased, if appropriate, or 1 or more agents of a different class of antihypertensive should be added.

3. If systolic BP ≥160 mmHg or diastolic BP ≥100 mmHg persists despite maximal antihypertensive therapy, then lenvatinib administration should be interrupted and restarted at 1 dose level reduction only when systolic BP ≤150 mmHg and diastolic BP ≤95 mmHg and the patient has been on a stable dose of antihypertensive medication for at least 48 hours.

- If systolic BP ≥160 mmHg or diastolic BP ≥100 mmHg recurs on the first dose reduction despite optimal management of hypertension with antihypertensive medications (either by dose increase or the addition of a different class of antihypertensive), then lenvatinib administration should be interrupted and restarted at an additional dose reduction only when systolic BP ≤150 mmHg and diastolic BP ≤95 mmHg and the patient has been on a stable dose of antihypertensive medication for at least 48 hours.
- If systolic BP ≥160 mmHg or diastolic BP ≥100 mmHg recurs on the second dose reduction despite optimal management of hypertension with antihypertensive medications (either by dose increase or the addition of a different class of antihypertensive), then lenvatinib administration should be interrupted and restarted at a third dose reduction dose only when systolic BP ≤150 mmHg and diastolic BP ≤95 mmHg and the patient has been on a stable dose of antihypertensive medication for at least 48 hours.

The following guidelines should be followed for the management of Grade 4 hypertension
(life threatening consequences):

1. Institute appropriate medical management

2. Discontinue drug

*Proteinuria:*

Regular assessment of proteinuria should be conducted. Grading according to CTCAE v4.03 should be based on the 24-hour urinary protein result if available. Management of lenvatinib administration should be based on the grade of proteinuria. In the event of nephrotic syndrome, lenvatinib must be discontinued. For detection and confirmation, a urine dipstick test should be performed. A 24-hour urine collection (initiated as soon as possible and at least within 72 hours) or an immediate spot urine protein-to-creatinine ratio (UPCR) test is required in the following situations:

- The first (initial) occurrence of ≥2+ proteinuria on urine dipstick while on treatment
- A subsequent increase in severity of urine dipstick proteinuria occurring on the same lenvatinib dose level
- When there has been a lenvatinib dose reduction and at the new dose level the urine protein dipstick result is ≥2+

A 24-hour urine collection should be initiated as soon as possible and at least within 72 hours to verify the grade of proteinuria when UPCR is ≥2.4.

Urine dipstick testing for patients with proteinuria ≥2+ should be performed as frequently as clinically indicated until the results have been 1+ or negative for 2 consecutive treatment cycles.

**References for Supplementary Appendix:**

1. Lenvima® (lenvatinib) [prescribing information]. Nutley, NJ, USA: Eisai Inc., 2021
2. Choueiri T et al. Poster presentation at ESMO. September 16–21, 2021. Poster #660P.
3. Motzer R, Alekseev B, Rha SY, et al. Lenvatinib plus pembrolizumab or everolimus for advanced renal cell carcinoma. N Engl J Med 2021;384:1289-1300.
4. Bianchi L, Rossi L, Tomao F, et al. Thyroid dysfunction and tyrosine kinase inhibitors in renal cell carcinoma. Endocr Relat Cancer 2013;20:R233-245.
